# Supplementary figures and images for: Full-length three-dimensional structure of the influenza A virus M1 protein and its organization into a matrix layer
Source: PLoS Biol. 2020 Sep 30;18(9):e3000827. doi: 10.1371/journal.pbio.3000827 (PMC7549809; doi:10.1371/journal.pbio.3000827)

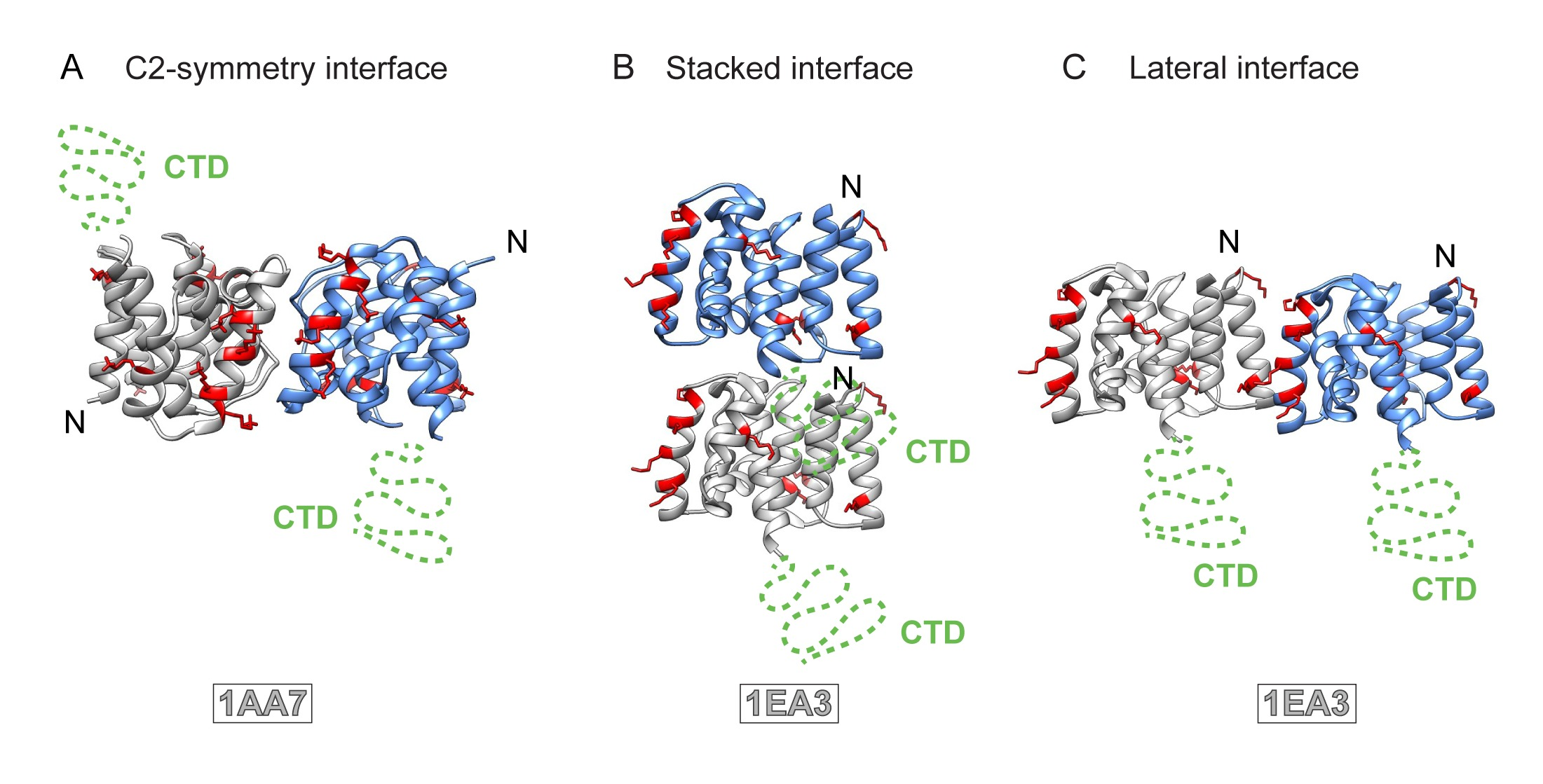

Supplement: S1 Fig — Shown are observed (A) C2-symmetry interface, present in structure PDB: 1AA7 [14], and (B) stacked and (C) lateral interfaces present in structure PDB: 1EA3 [15]. Lysine residues, used below in crosslinking analyses, are shown in red. Hypothetical locations of CTDs are shown in green. CTD, C-terminal domain; M1, matrix protein 1; NTD, N-terminal domain; PDB, Protein Data Bank (TIF) [file pbio.3000827.s001.tif]

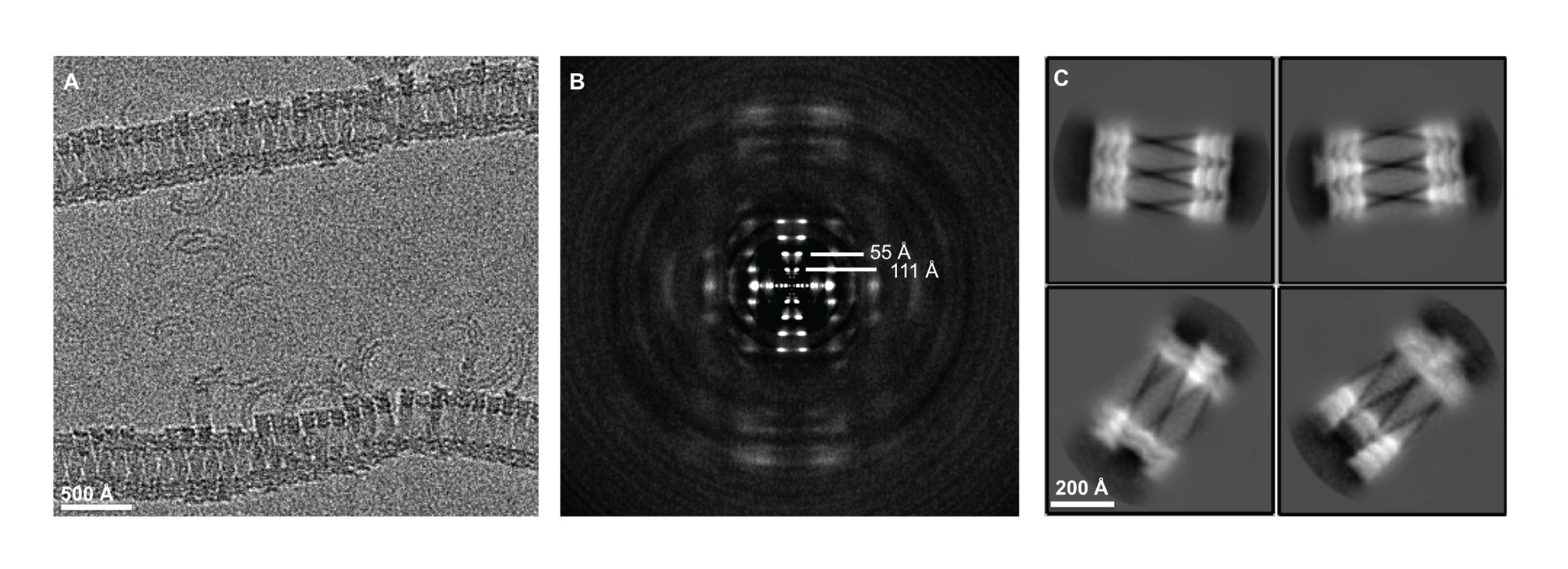

Supplement: S2 Fig — (A) Representative cryo-EM micrograph of WT-M1 oligomers. (B) Fourier transform of the image in (A) shows layer lines. The first layer line indicates the 111-Å helical pitch of the WT-M1 oligomer. (C) 2D class averages that focus on the center of the helical segments show large heterogeneity of additional density on either side of the outer layer of the filament. cryo-EM, cryo-electron microscopy; M1, matrix protein 1; WT-M1, full-length PR8 M1. (TIF) [file pbio.3000827.s002.tif]

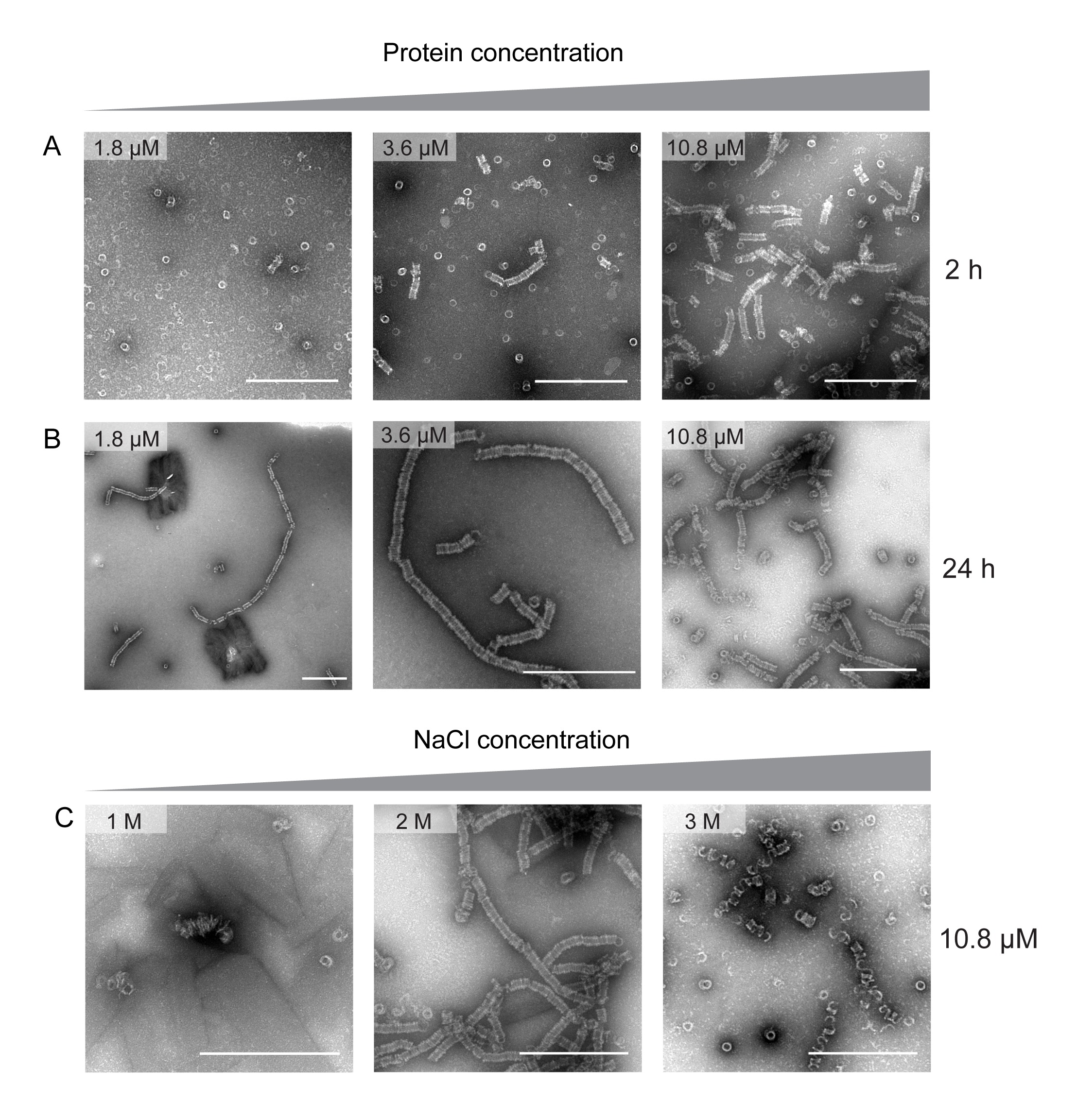

Supplement: S3 Fig — Negative-stain electron micrographs (A) after 2 h and (B) 24 h of incubation at the indicated concentrations of protein in the presence of 2 M NaCl. (C) Negative-stain electron micrographs following 24-h incubations of 10.8 μM M1 assembled at NaCl concentrations indicated. Scale bar = 5,000 Å. M1, matrix protein 1 (TIF) [file pbio.3000827.s003.tif]

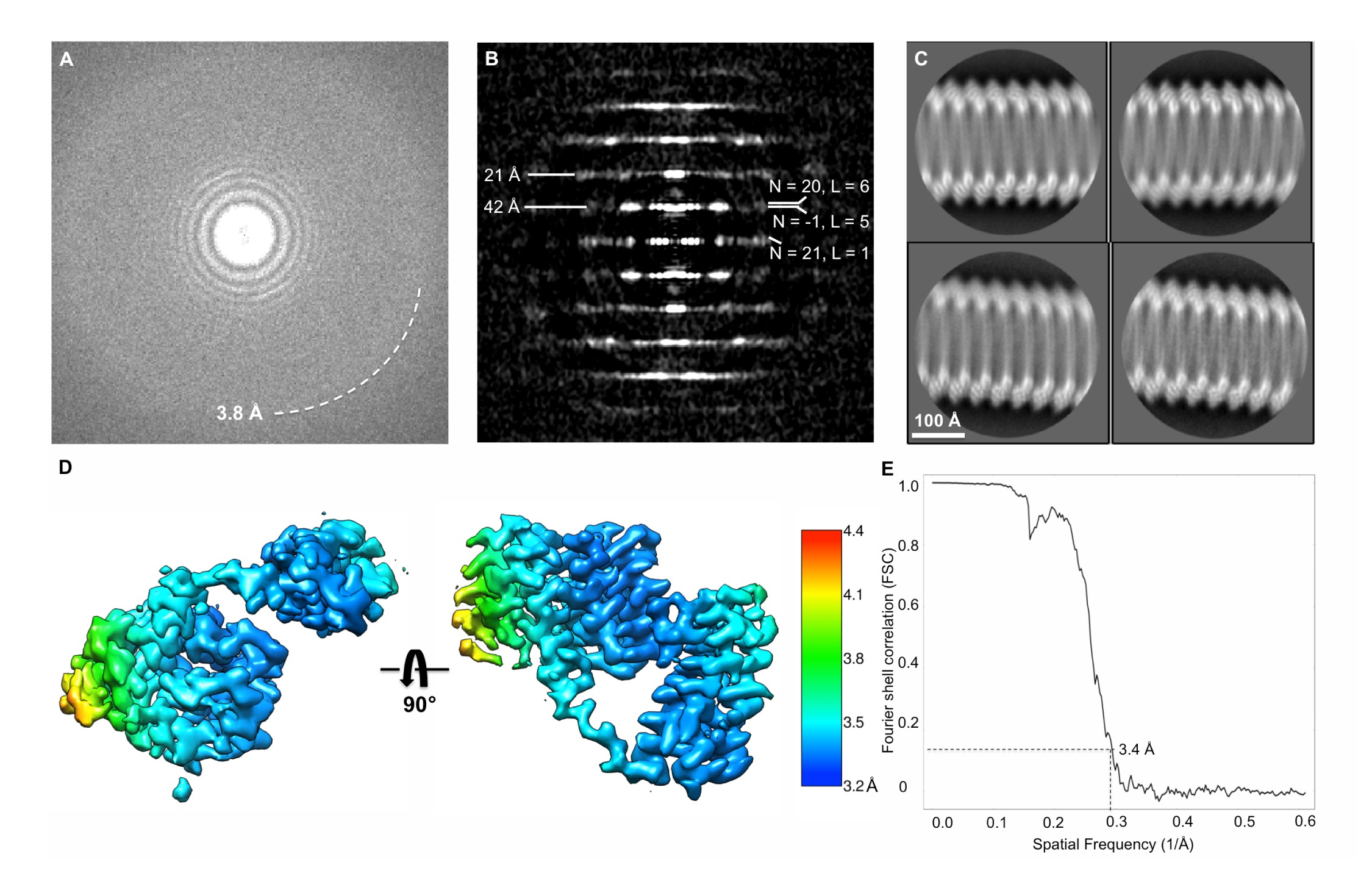

Supplement: S4 Fig — (A) Fourier transform of a representative micrograph that shows the water ring. (B) Layer line indexing. (C) Representative 2D class averages. (D) Local resolution of the M1-V97K asymmetric unit. (E) FSC curve shows 3.4 Å resolution using the 0.143 cutoff. cryo-EM, cryo-electron microscopy; FSC, Fourier shell correlation; M1, matrix protein 1 (TIF) [file pbio.3000827.s004.tif]

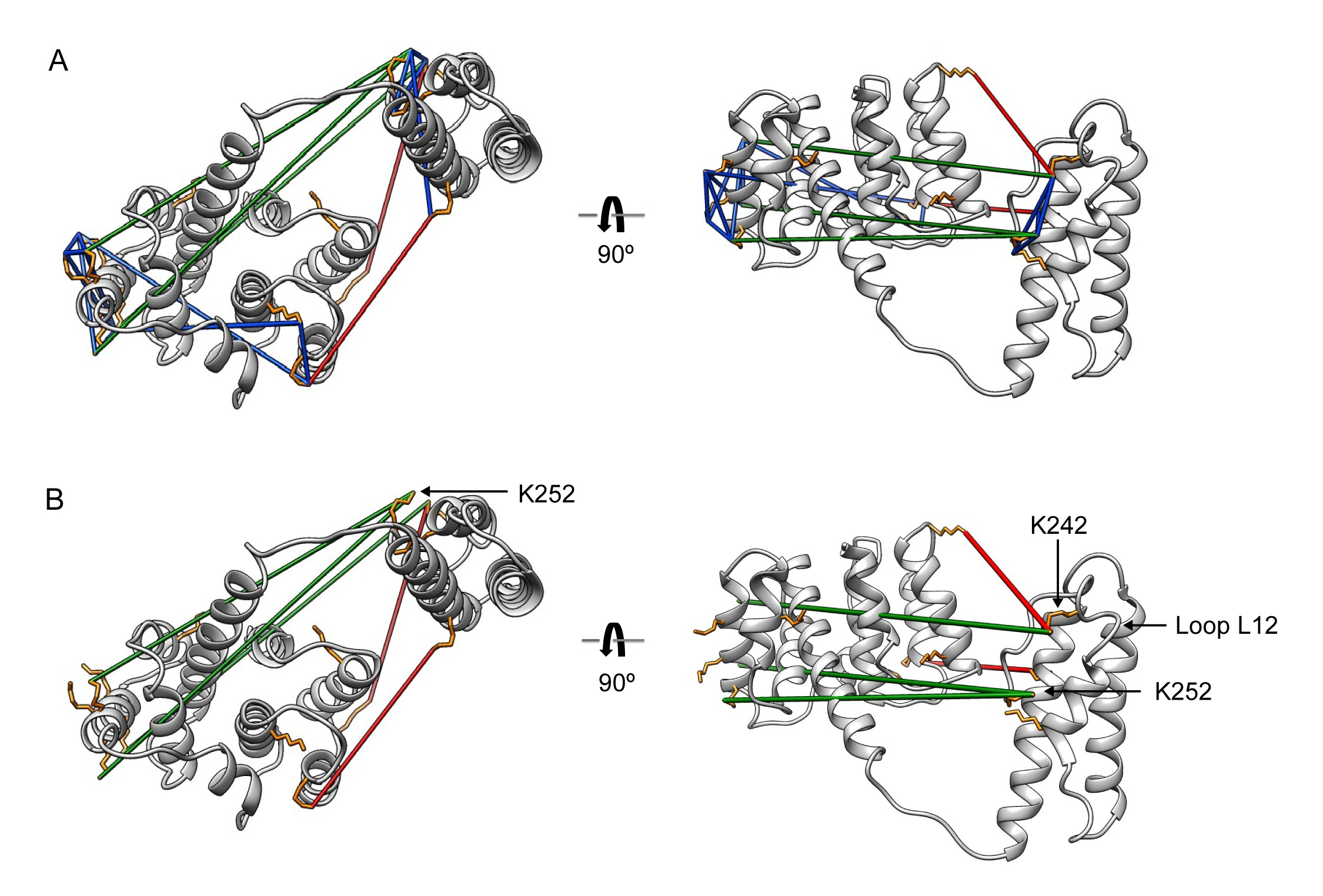

Supplement: S5 Fig — (A) Ribbon representation of one M1-V97K protein in the same orientations as shown in Fig 5, displaying the discovered crosslinks applying the same color scheme as in Fig 4. Lysine residues are shown as orange sticks. (B) Same as in (A), except only green and red crosslinks are displayed to highlight crosslinks formed by lysine K242 and K252, located on flexible loop L12. M1, matrix protein 1 (TIF) [file pbio.3000827.s005.tif]

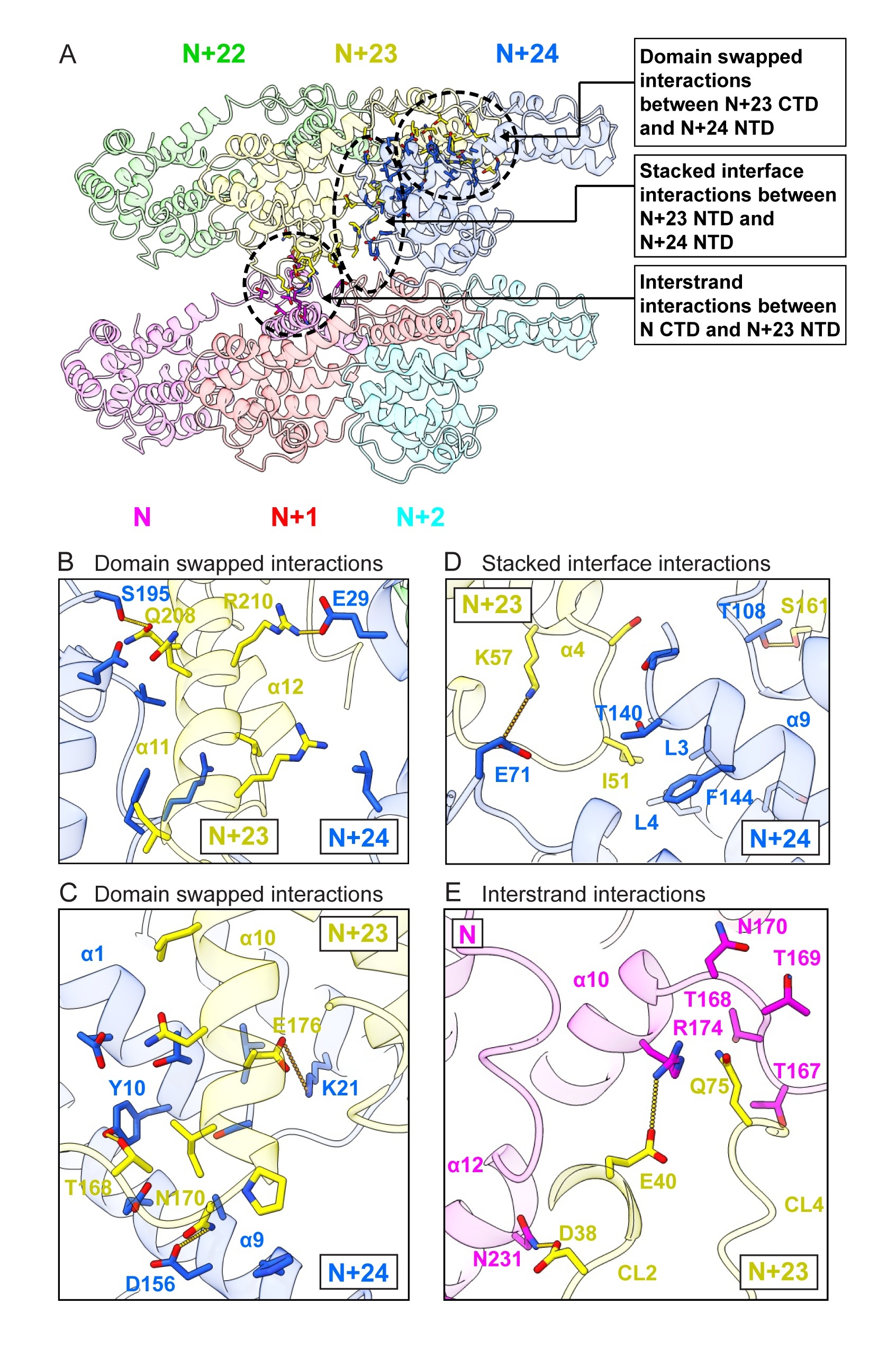

Supplement: S6 Fig — (A) A group of 6 asymmetric units with the lower strand identified as N (pink), N + 1 (red), and N + 2 (cyan) and the upper strand identified as N + 22 (green), N + 23 (yellow), and N + 24 (blue). Dotted circles highlight 3 groups of intermolecular interactions. (B) Interactions between N CTD and N + 23 NTD at the interstrand interface. Five residues T167–169, N170, and R174 from N participate in a hydrophobic pocket that also contains Q75 from N + 23. (C) Interactions between NTDs of 2 adjacent asymmetric units N + 23 and N + 24. Residues L3, L4, T140, and F144 from N + 24 form a hydrophobic pocket with I51 from N + 23. (D) Connecting loop CL9 and helix α10 from the CTD of N + 23 interact with N + 24 NTD via hydrogen bonds and hydrophobic interactions. (E) Helices α11 and α12 from the CTD of N + 23 interact with N + 24 via hydrogen bonds, salt bridges, and hydrophobic interactions. Yellow dashed lines indicate hydrogen bonds and salt bridges. Orientations are altered to facilitate visualization. CL, connecting loop; cryo-EM, cryo-electron microscopy; CTD, C-terminal domain; M1, matrix protein 1; NTD, N-terminal domain. (TIF) [file pbio.3000827.s006.tif]

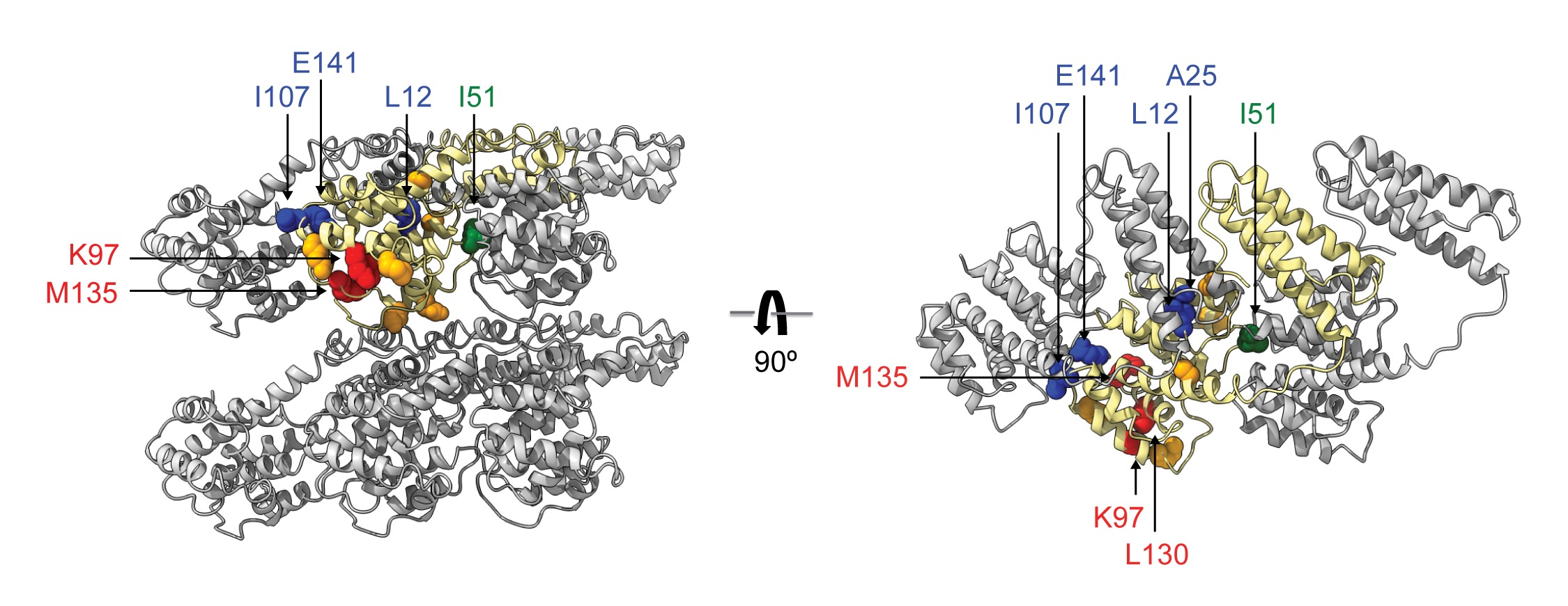

Supplement: S7 Fig — All residues mutated in the WT-M1 background are displayed as spheres in the context of the M1-V97K oligomer. Mutated residues that resulted in single-layered, no apparent, or multilayered oligomerization are displayed as red, blue, and orange spheres, respectively. Residue I51, at which mutations resulted in no apparent or multilayered oligomerization, is displayed in green. M1, matrix protein 1; WT-M1, full-length PR8 M1. (TIF) [file pbio.3000827.s007.tif]

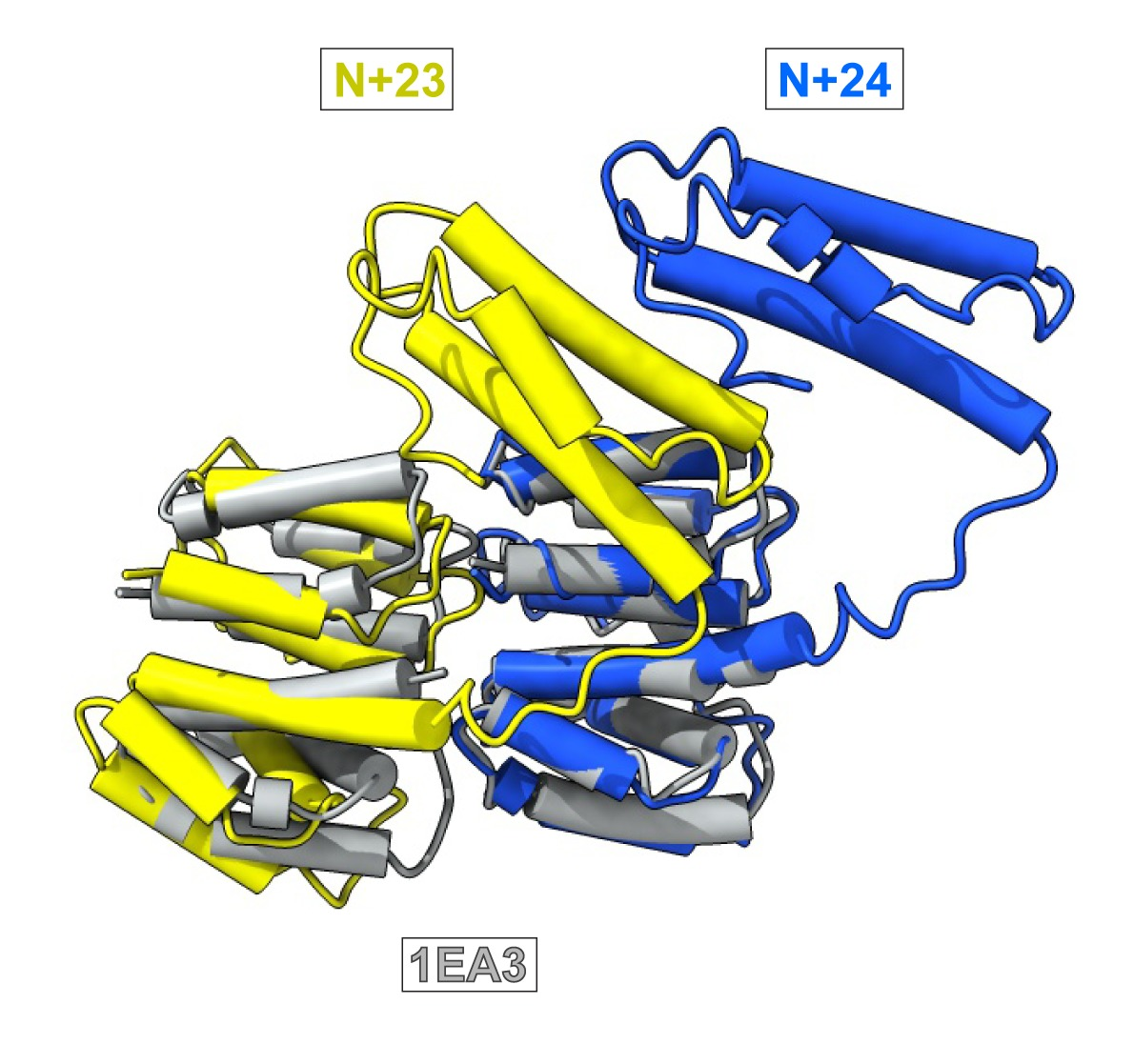

Supplement: S8 Fig — Comparison of the stacked interface between 2 subunits (gray) of the crystal structure of NTDM1 (PDB: 1EA3) [15] and the NTDs of 2 M1 subunits, N + 23 (yellow) and N + 24 (blue), of the cryo-EM structure of M1-V97K oligomers. The lack of superposition of the yellow and leftmost gray subunit reveals the slight alteration in the stacked interface between the NTD and full-length M1 structures. cryo-EM, cryo-electron microscopy; M1, matrix protein 1; NTD, N-terminal domain; PDB, Protein Data Bank (TIF) [file pbio.3000827.s008.tif]

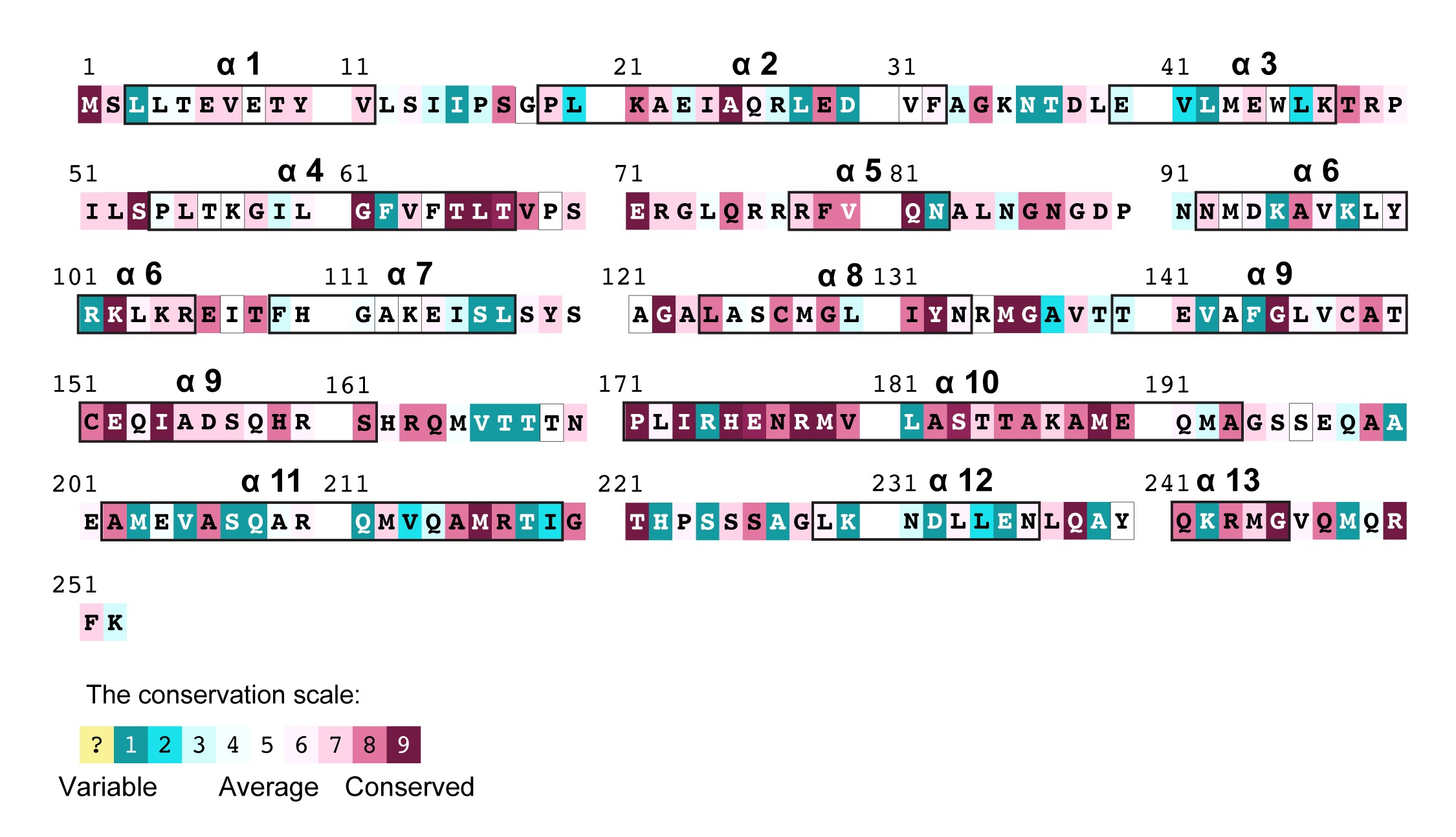

Supplement: S9 Fig — Amino acids are shown with each residue colored by its conservation score, as calculated using the ConSurf server. M1, matrix protein 1 (TIF) [file pbio.3000827.s009.tif]

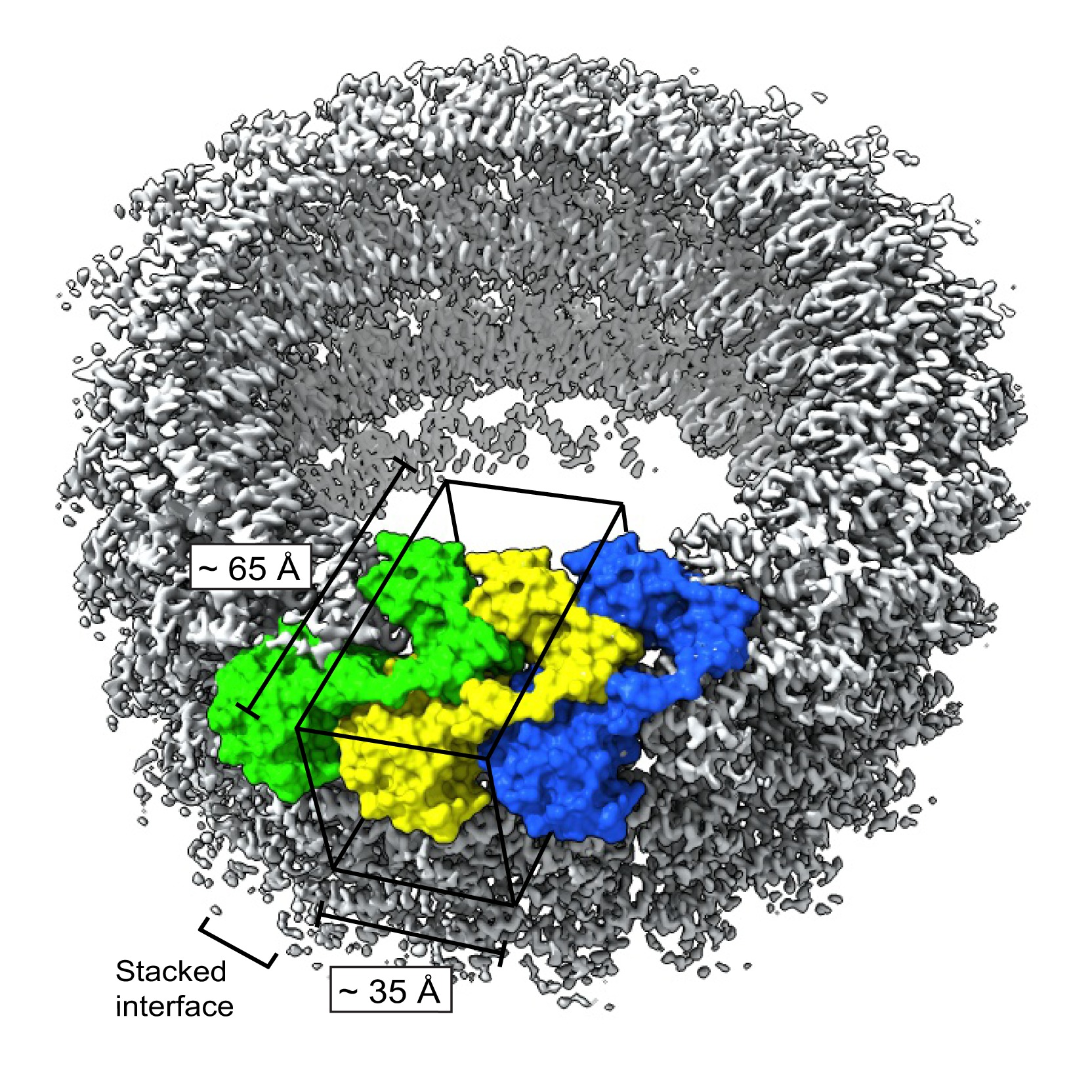

Supplement: S10 Fig — Shown are 3 adjacent subunits within the M1-V97K tube with the stacked interface between M1 monomers indicated. A box highlights the length and width of the yellow subunit. The NTD is pointed towards the outside of the tube. M1, matrix protein 1; NTD, N-terminal domain. (TIF) [file pbio.3000827.s010.tif]

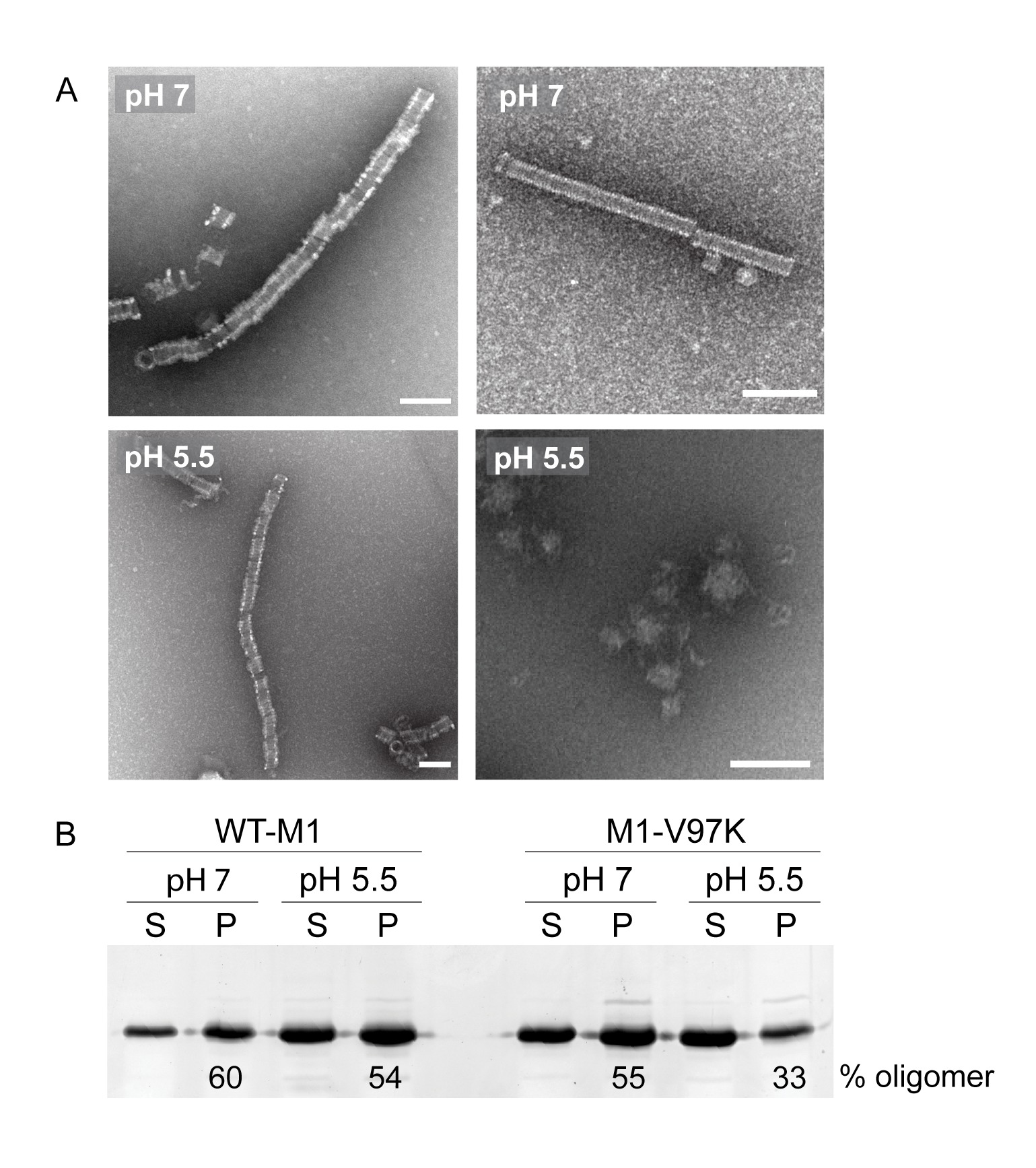

Supplement: S11 Fig — (A) Negative-stain electron micrographs of WT-M1 (left) and V97K-M1 (right) oligomers that were pelleted and resuspended in buffer at pH 7 or 5.5 as indicated. (B) SDS-PAGE quantification of pellets (P) and supernatants (S) after resuspending WT-M1 and M1-V97K oligomers in buffer at pH 7 or 5.5. M1, matrix protein 1; WT-M1, full-length PR8 M1. (TIF) [file pbio.3000827.s011.tif]

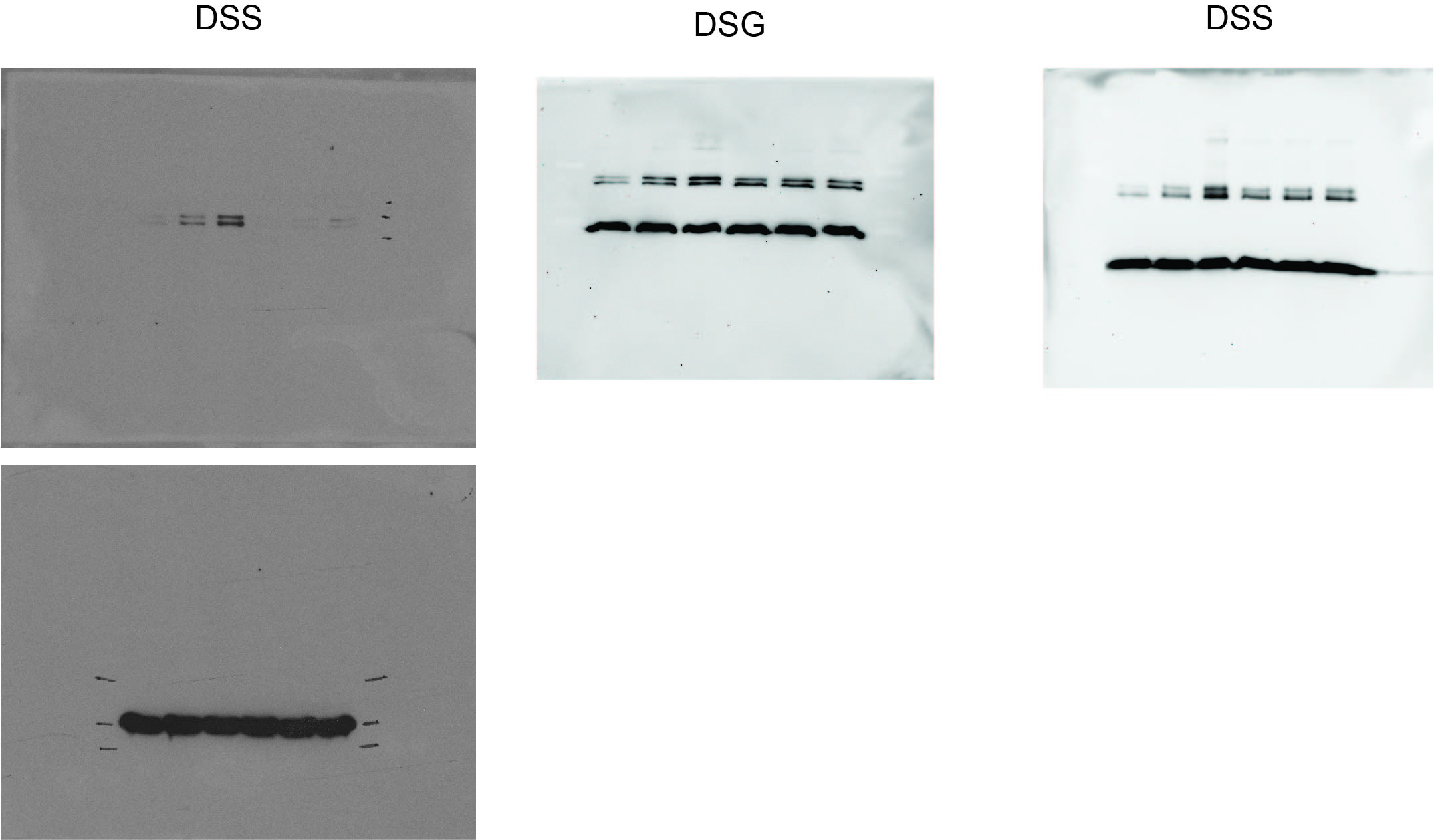

Supplement: S1 Raw Images — (JPG) [file pbio.3000827.s022.jpg]
