# Supplementary material for: Full-length three-dimensional structure of the influenza A virus M1 protein and its organization into a matrix layer
Source: PLoS Biol. 2020 Sep 30;18(9):e3000827. doi: 10.1371/journal.pbio.3000827 (PMC7549809; doi:10.1371/journal.pbio.3000827)
Supplement: S1 Table — IAV, influenza A virus; M1, matrix protein 1; NTD, N-terminal domain; PDB, Protein Data Bank (DOCX) [file pbio.3000827.s012.docx]

**S1 Table. Protein-protein interfaces and crystallization conditions of all IAV NTD^M1^ structures deposited to the protein data bank at rcsb.org**

| PDB | pH | C2-symmetry interface | Stacked interface | Lateral interface | No interface | Asymmetric unit |
| --- | --- | --- | --- | --- | --- | --- |
|  |  | 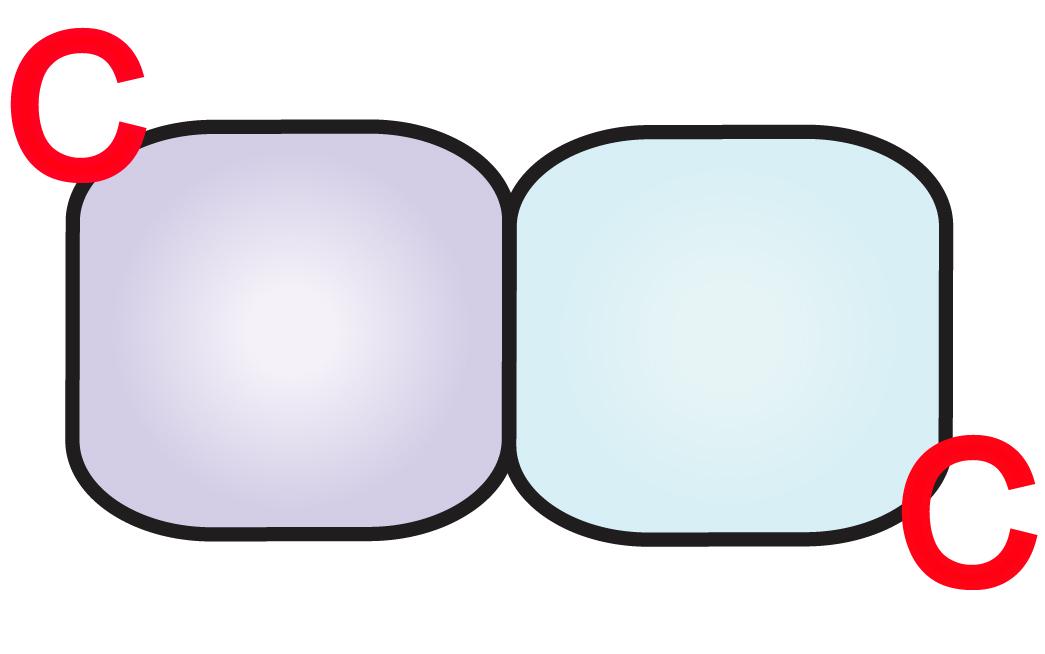 | 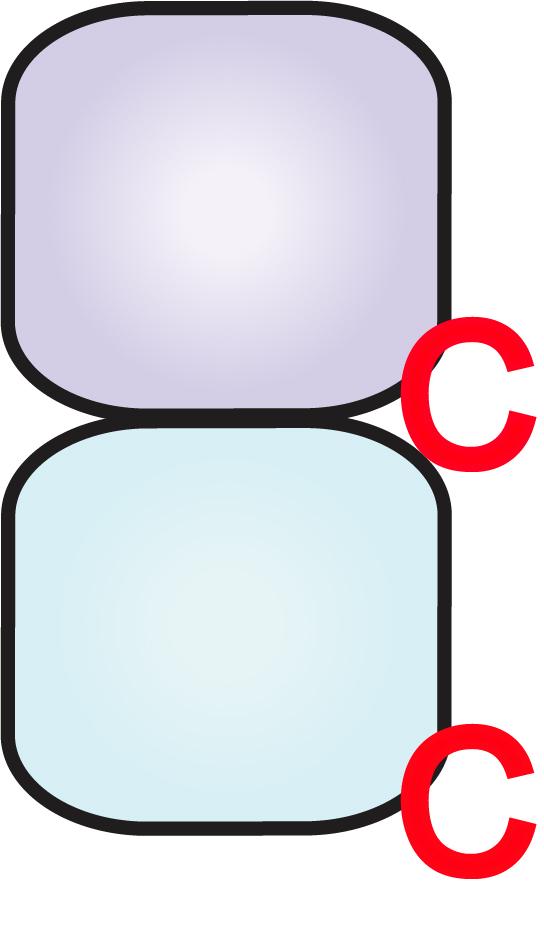 | 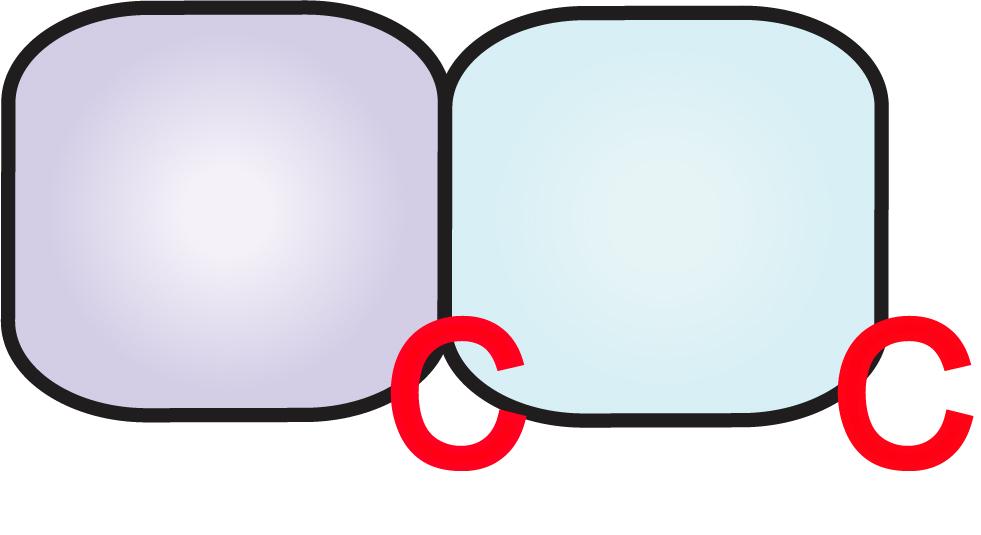 |  |  |
| 2Z16 | 8 |  |  |  | 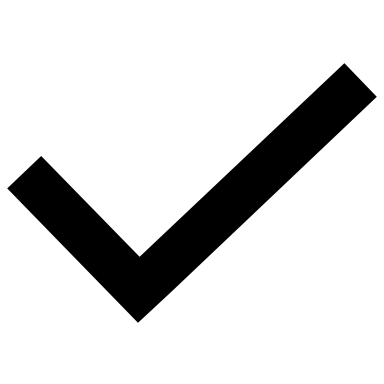 | 2 monomers |
| 6I3H (G18A) | 7.5 |  |  | 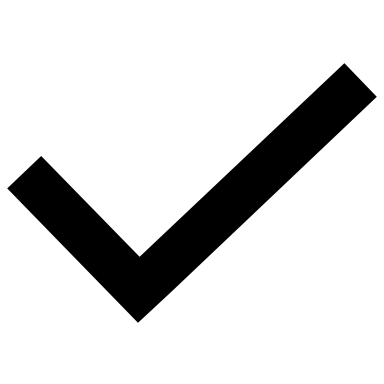 |  | 2 monomers |
| 5V8A (G88R) | 7.3 |  | 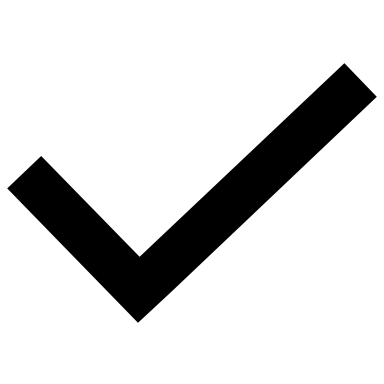 | 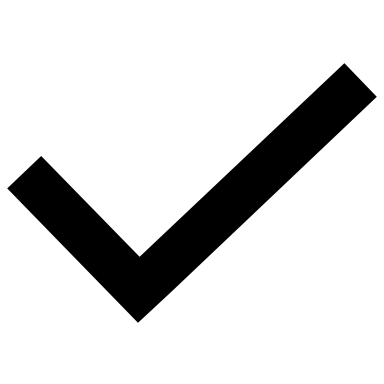 |  | 1 monomer |
| 1EA3 | 7 |  | 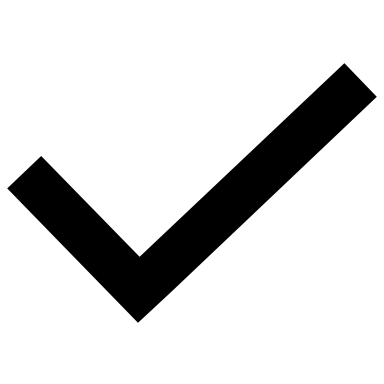 | 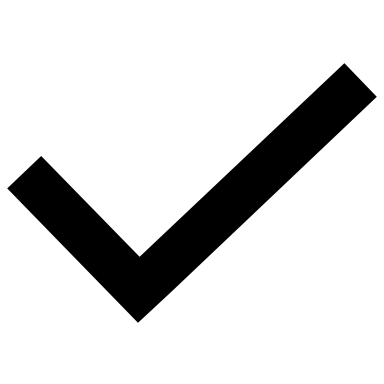 |  | Stacked dimer |
| 3MD2 | 7 | 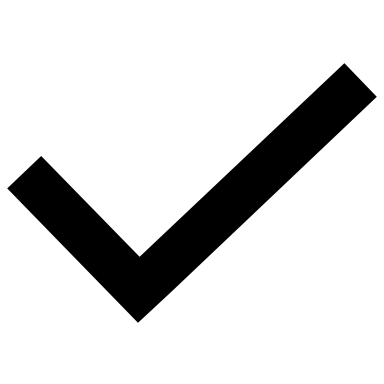 | 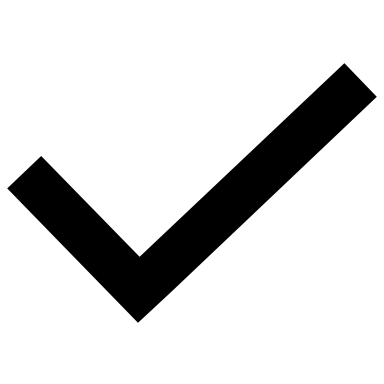 |  |  | C2-symmetry dimer |
| 5V7B (G88E) | 7 | 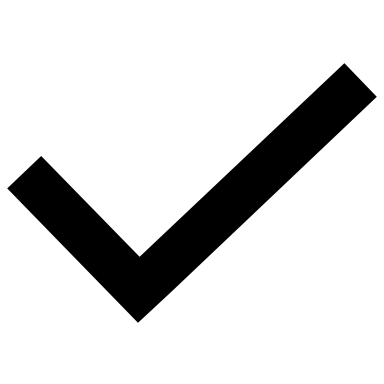 |  |  |  | C2-symmetry dimer |
| 5V7S (G88E) | 6.2 | 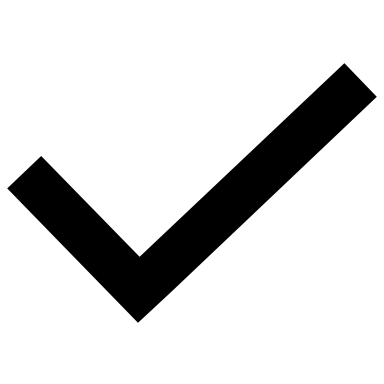 | 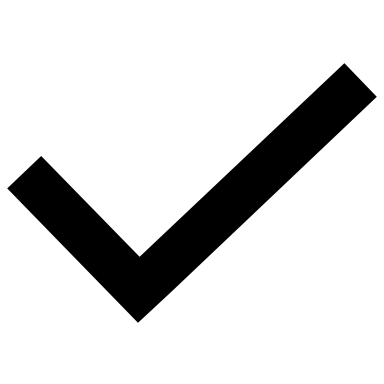 |  |  | Trimer with C2-symmetry and stacked interface |
| 5V6G (G88R) | 5.5 | 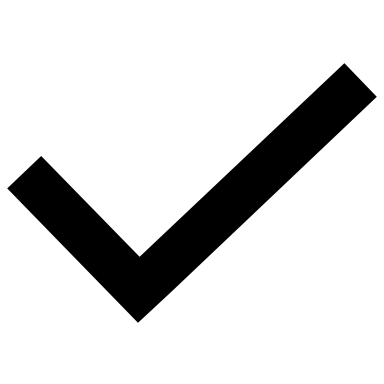 | 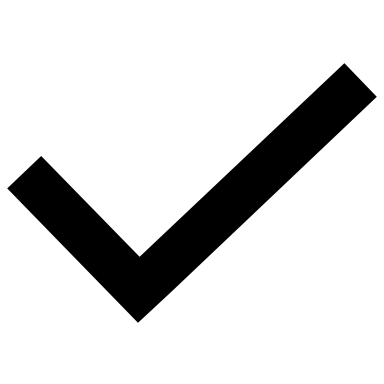 |  |  | Trimer with C2-symmetry dimer Stacked dimer |
| 5CQE | 5 | 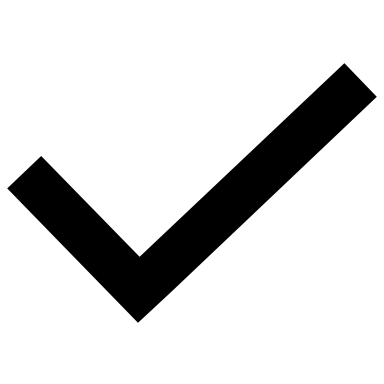 | 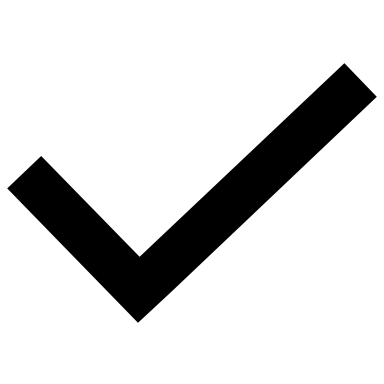 |  |  | C2-symmetry dimer |
| 4PUS | 4.7 | 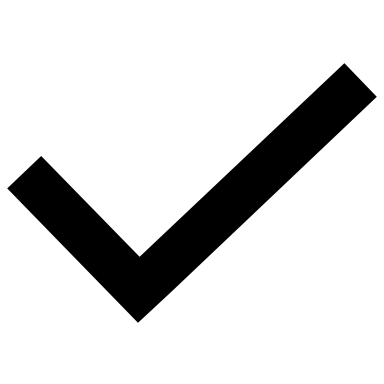 |  |  |  | C2-symmetry dimer |
| 1AA7 | 4 | 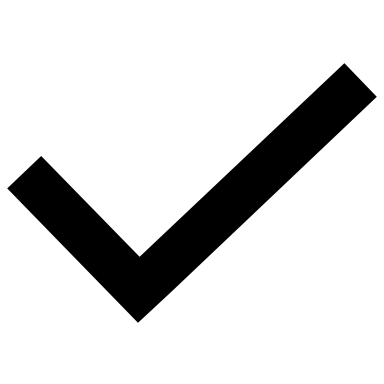 | 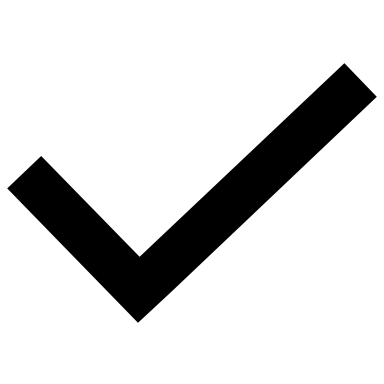 |  |  | C2-symmetry dimer |
