# Supplementary material for: Full-length three-dimensional structure of the influenza A virus M1 protein and its organization into a matrix layer
Source: PLoS Biol. 2020 Sep 30;18(9):e3000827. doi: 10.1371/journal.pbio.3000827 (PMC7549809; doi:10.1371/journal.pbio.3000827)
Supplement: S2 Table — Mutations at the stacked, lateral, and C2-dimer interface. (DOCX) [file pbio.3000827.s013.docx]

**S2 Table. Mutations at the stacked, lateral and C2-dimer interface**

|  | **Mutation** | **Location** |
| --- | --- | --- |
| **Single-layered** | | |
| Non-conservative | V97K | C2 symmetry or Lateral interface |
|  | L130Q/M135Q | C2 symmetry or Lateral interface |
| **No apparent assembly** | | |
| Non-conservative | L12K | Lateral interface |
|  | A25K | Lateral interface |
|  | I51W | Stacked interface |
|  | I107K | Stacked interface |
|  | E141A | Stacked interface |
|  | E141G | Stacked interface |
|  | E141I | Stacked interface |
|  | E141K | Stacked interface |
|  | E141R | Stacked interface |
|  | I51E/E141I | Stacked interface |
| Conservative | I107G | Stacked interface |
| **Multi-layered** | | |
| Non-conservative | G18K | Lateral interface |
|  | A22K | Lateral interface |
|  | E40R |  |
|  | E44R |  |
|  | I51E | Stacked interface |
|  | I51K | Stacked interface |
|  | M93Q | C2 symmetry or Lateral interface |
|  | K104N | C2 symmetry or Lateral interface |
| Conservative | I51G | Stacked interface |
|  | A155G |  |
|  |  |  |
