# Supplementary material for: Full-length three-dimensional structure of the influenza A virus M1 protein and its organization into a matrix layer
Source: PLoS Biol. 2020 Sep 30;18(9):e3000827. doi: 10.1371/journal.pbio.3000827 (PMC7549809; doi:10.1371/journal.pbio.3000827)
Supplement: S3 Table — Cryo-EM data collection, processing, and model refinement statistics of the WT-M1 and M1-V97K M1 filaments. cryo-EM, cryo-electron microscopy; M1, matrix protein 1; WT-M1, full-length PR8 M1. (DOCX) [file pbio.3000827.s014.docx]

**S3 Table. Cryo-EM data collection, processing, and model refinement statistics of the WT-M1 and M1-V97K M1 filaments.**

| **Cryo-EM data collection and processing** | M1-WT | M1-V97K |
| --- | --- | --- |
| Microscope | Tecnai F20 | Titan Krios |
| Voltage (kV) | 200 | 300 |
| GIF Quantum energy filter slit width (eV) | N.A. | 20 |
| Detector | Gatan K2 | Gatan K2 Bioquantum |
| Magnification | 14,500 X | 165,000 X |
| Pixel size (Å) | 2.53 | 0.82 |
| Symmetry imposed | C1 | C1 |
| Defocus range (μm) | -0.8 – - 4.2 | - 0.3 – - 4.3 |
| Electron exposure (e^-^/Å^2^) | 12 | 36 |
| Micrographs (acquired/used) | 100/100 | 440/435 |
| Segment step (asymmetric unit) | N.A. | 5 |
| Number of extracted particles/asymmetric units | 54,585/N.A. | 59,928/299,640 |
| Number of particles after 2D classifications | 53,880/N.A. | 56,602/283,010 |
| Helical twist and rise (^°^/Å) | N.A. | 17.1/1.96 |
| Map resolution at 0.143 FSC criterion (Å) | N.A. | 3.4 |
| Local resolution range (Å) | N.A. | 3.3-4.3 |
| Sharpening B-factor (Å) | N.A. | -96 |
|  |  |  |
| **Model refinement** |  | 9 asymmetric units |
| Atoms | N.A. | 23,403 |
| Residues | N.A. | 2,259 |
| CC_mask_ | N.A. | 0.73 |
| Resolution_FSC map vs. model @ 0.5_ (Å) | N.A. | 3.4 |
| Ramachandran plot (%) |  |  |
| Outliers | N.A. | 0.4 |
| Allowed | N.A. | 5.22 |
| Favored | N.A. | 94.38 |
| Rotamer outliers (%) | N.A. | 5.74 |
| r.m.s. deviations |  |  |
| Bond lengths (Å) | N.A. | 0.007 |
| Bond angles (°) | N.A. | 0.990 |
| MolProbity score | N.A. | 2.17 |
| Clash score | N.A. | 4.28 |
| Q-score | N.A. | 0.59 |
| Q-score expected at 3.4Å | N.A. | 0.52 |
