# Supplementary material for: Full-length three-dimensional structure of the influenza A virus M1 protein and its organization into a matrix layer
Source: PLoS Biol. 2020 Sep 30;18(9):e3000827. doi: 10.1371/journal.pbio.3000827 (PMC7549809; doi:10.1371/journal.pbio.3000827)
Supplement: Attachment — Submitted filename: Selzer responses 8.17.docx [file pbio.3000827.s023.docx]

**Responses to reviewers:**

**Reviewer #1:**

**1.** *“A key part of the narrative is the demonstration by cross-link mass-spectrometry that the structure of the V97K single layer tubes mimics the organisation in the neutral pH virions and is different from the low pH, multi-layer situation. The 'red' cross-links in the figure are consistent with this and consistent with the structure. However the 'green' cross-links are consistently too long in the structure. Can the authors give a better explanation of this?*

Yes, the green crosslinks (50-54 Å) initially seem long for a crosslinker of 11.4 Å! We have added to this discussion a better description of potential contributions to this distance. We have also updated Figure S5 to highlight the flexible loop region L12 in the C-terminal domain that contains two of the crosslinked lysines.

See lines 231-237:

“All previously determined crosslinks mapped well to the M1-V97K cryo-EM structure, showing crosslinking distances between 5-40 Å that are consistent with distances reported in the literature [23] (Fig 5C, S5 Fig). The crosslinks depicted in green, formed between NTD lysine residues K95 and K97 and CTD residues K242 and K252 on the CTD, are predicted by the structure to crosslink distances of 50-54 Å. This can be attributed to the length and side chain rotations of the lysine residues, which point in opposite directions in the solved structure, and the conformational flexibility of the C-terminal loop region L12 (S5 Fig).”

.”

**2.** ”*It is not clear to me why the authors assume that all detected cross-links are intramolecular, since there are clearly some dimers in Figure 4B (which is not referred to in the text). Could this be an explanation*?”

Thank you for giving us the opportunity to clarify this - we only analyzed proteins that ran as monomers by SDS PAGE to investigate intramolecular crosslinks. We added a better description of this in the manuscript, on lines 196-202:

“To monitor any alterations in M1 conformation under the various conditions, crosslinking of PR8 virions, Udorn virions, purified WT-M1 oligomers and purified M1-V97K oligomers was performed by incubation with DSS. The extents of crosslinking were inferred from the proportion of M1 dimerization observed in virions (Fig 4A) and in purified protein oligomers (Fig 4B). Crosslinking conditions were optimized to yield theoretical frequencies of fewer than one per crosslink per monomer. To ensure that only intramolecular crosslinks were analyzed, monomeric bands from samples separated by SDS-PAGE were excised”.

**3.** *“Would the structure predict any inter-molecular cross-links?”*

Yes, the structure predicts many inter-molecular crosslinks and these would indeed be interesting to analyze. We chose to analyze the results obtained at a low frequency of crosslinking in hopes of better sampling independent events. At this frequency, covalent dimers were not sufficiently abundant for analysis, especially for the virion samples – presumably due to the presence of many other proteins as well. We hope the changes described above and on lines 194-202 help to clarify this point.

4. Have the authors any idea how a single layer could morph into a multilayer and whether this would require significant conformational changes in the protein?

Yes, and thank you for the opportunity to speculate, which we now do in the Discussion. We hypothesize that the alteration from the single-layer conformation, in which the NTD and CTD are oriented more closely to each other, occurs via a change in angle between these domains. We have added a better description of this in the manuscript. Specifically, in lines 369-382:

“the similarities in ultrastructure and crosslinking patterns between oligomers of WT full-length M1 and disrupted virions argue that the WT-M1 oligomers represent the collapsed, lower-energy state. Conversion of the single-layer matrix in infections into the multi-layer state during cell entry is likely due to a pH or metal-ion trigger within the endosome [6,38]. We hypothesize that protonation of the histidine cluster (Fig 6G and Fig 6H) in infectious virions introduces repulsive forces that alter the orientation of the CTD relative to the NTD within each M1 monomer. Indeed, it has been shown that a peptide derived from the C9 linker sequence, which connects the NTD and CTD (Fig 5C) and contains two of the five histidine residues, folds into an alpha helix at low pH [37]. Subsequent detachment of the matrix layer from the viral envelope would then expose the Val 97-containing M1 surface, allowing the assembly of further M1 subunits and giving rise to multi-layered oligomers (Fig 7). Subsequent to this step, it is likely that the M1 oligomers from disrupted virions disassociate; studies using fluorescence microscopy of infected cells have shown ubiquitination and dispersion of M1 into the cytoplasm after endosomal fusion [11,12].”

**5.** “*The outer surface is positively charged consistent with membrane binding. Can the authors comment on how this surface might also bind the glycoprotein trans-membrance tails*?”

We thank the reviewer for this question. Yes, there does need to be some specificity for the proteins as well. We have added some comments to the Discussion on lines 335-344:

“If this is the case, it is likely that the positively charged outside of the single-shelled M1-V97K tube is adjacent to the negatively charged viral membrane and the cytosolic portions of the glycoproteins [34]. The cytosolic tail of hemagglutinin has been shown to be palmitoylated, which, in addition to other, as yet unidentified intermolecular contacts, could mediate the interactions with M1 through its negative charge [35].”

**6.** “*The inner surface is negatively charged. Can the authors comment on how this surface might interact with RNPs*?”

Yes, it is interesting because the RNP complexes display patches of both positive and negative charge, This is discussed, and an excellent reference added, on lines and there is an excellent reference for this. to which we now refer (and give a reference) on Lines 355-367:

“These observations support the hypothesis that NTDs of the single-layered influenza M1 oligomers, represented by the high-resolution structure of the M1-V97K oligomers reported here, face the negatively charged viral membrane and the cytoplasmic domains of the viral glycoproteins embedded within [35]. For example, the cytosolic tail of IAV hemagglutinin is known to be palmitoylated, which could contribute to M1 interactions through its negative charge [36]. On the inside of the IAV matrix layer, negatively charged regions of the CTDs are likely to contact the positively charged patches on the ribonucleoprotein complexes [37].”

**Reviewer #2:**

**1.** “*The Discussion section provides a particularly nice mini-review and analysis that puts the current work into context of what has previously been published…..Overall the biochemistry and cryoEM studies and model refinement are well performed and the conclusions are consistent with the data. In fact it is difficult to find any flaws in this manuscript. Kudos*.”

Thank you so much!

**2.** “*Fig 6 the colors are not exactly consistent. For example in panels C/D the green is different than in panels G/I.”*

We thank the reviewer for pointing this out. We have changed the subunit colors, as making them more transparent proved to be more confusing than helpful.

**3.** “*Fig S5 could be moved to Figure 4 to provide better structural context for the crosslinking MS*.”

We agree that the superposition of the crosslinks found in Figure 4 onto the structure found in Figure 5 is very interesting, and that Figure 4 makes one curious about them. However, given the flow of the narrative, we did not want to anticipate discussion of the cryo-EM structure shown in Figure 5 with the discussion of crosslinking in Figure 4 before the reader knows why the structural data are convincing. Instead, the crosslinks and the relevant distances are shown in Figure S5, and discussed in the text on lines 231-237.

**Reviewer #3:**

**1.** “*This is a very nice paper presenting long-awaited data of a high-resolution structure of the influenza matrix protein assembled into a polymer attached to membranes. The work appears to be carefully done and the findings are likely to be high impact. I raise a few questions below, mostly related to data interpretation or presentation*.”

Thank you so much!

**2.** “*The use of 2-3M NaCl to induce M1 oligomerisation is surprising but the resulting oligomeric structures fit well into existing knowledge of the M1 oligomer. Is there a precedent for the use of high salt in such situations or a physiological rationale*?”

Yes, this is a trick used in the capsid-assembly field. We have added a description in the manuscript to explain this better, and to provide specific references, on lines 292-294.

“High ionic strength has been widely used for oligomerization of viral capsid proteins, such as those of HIV and hepatitis B virus, due to its effects in neutralizing charges between protein subunits [31,32].“

**3.** “*The predominance of multilayering is somewhat unexpected. Is it possible that this arises from having an empty particle, and that in a virion a second or third layer would be prevented due to interactions of M1 with RNPs or other components*?”

It was indeed an attractive hypothesis that the multilayering could be shielded by the RNPs, and thus perhaps be a feature of packaging. However, it is not consistent with the location of the Val97 residue required for forming the multiple layers. Val97 is on the outside of the filament, which is argued to be facing the membranes. The asterisked reference for the 8 Å structure of intact influenza virions was in June 2020 Biorxiv. The proposed orientation of the M1 matrix layer in virions is now discussed in more detail in lines 267- 271.

“In infectious salmon anemia virus particles, the NTDs of M1 contact the viral envelope, an orientation that has also been suggested by the very recent cryo-electron tomography of filamentous influenza A particles at 8 Å resolution [34]*. These observations support the hypothesis that the NTDs of the single-layered influenza M1 oligomers, represented by the high-resolution structure of the M1-V97K oligomers reported here, face the negatively charged viral membrane and the cytoplasmic domains of the viral glycoproteins embedded within [35].“

**4**. “*The work very nicely includes a large amount of mutagenesis, however perhaps this could be taken better advantage of*”.

Thank you, and we agree. Anyone one of those mutations could turn into its own project and it has been very hard to stop.

**5.** Three questions related to the mutations:

“(1) *L130 /M135Q mutant, the micrograph for this mutant looks very disorganised and quite different from the representative particle shown for V97K. Is the disorganisation or perhaps relative amounts of single-layered and multi-layered oligomerisation something that can be quantified?*”

We too are interested in these mutations. It does appear that only short regions of the L130/M135Q mutant tubes shown in Figure 3 are structurally similar to those of V97K. The extent to which the L130/M135Q tubes resemble the V97K tubes is highly variable between tubes, as though the formation of multilayered oligomers is a cooperative process. Quantitation of this would, indeed, be interesting, but has proved very challenging given the variability.

**6.** “*On page 8, second paragraph it is suggested that L12 and A25 are at the stacked dimer interface but this doesn't seem to be the case. They are, interestingly, packed against one another on a lateral side of the protein. Does the polymeric structure suggest they are important in stabilising NTD-CTD interactions? Crosslinking suggests a contact between a-helix 2 of the NTD and the CTD, although some (irregular) assembly seems to happen just with the NTD alone*.”

We thank the reviewer for pointing out this mistake. We have corrected the text in the manuscript on lines 160-165:

“Most mutations in the stacked interface, such as residue I51 into tryptophan, residue I107 into glycine or lysine, or any mutation at residue E141, prevented the assembly of any oligomers (Fig 3B, S2 Table). Interestingly, mutations at residue L12 and A25 into lysine at the lateral interface also resulted in no apparent oligomerization. This argues that hydrophobic and electrostatic interactions at the stacked and lateral interface, especially residue 141, are required for oligomerization of M1.”

And in lines 264-268:

“In addition to the stacked interface neighboring subunits interact via NTD-CTD interactions. Two residues, L12 and A25, which are located at the lateral interface within the crystal structure of NTD^M1^ are form hydrophobic interactions at the NTD-CTD interface within M1-V97K oligomers (S7 Fig). Consistent with this, mutations of L12 and A25 to lysine prevented oligomerization of M1.”

**7.** “*Related to the above point, it seems like it would be more useful to map the mutation sites onto a subunit within the polymeric structure that was determined to see if there effects (or lack of) can be understood?”*

We agree with the reviewer that it is useful to see the mutations mapped to the oligomeric structure of V97K as well. We made an additional supplementary figure (S Fig7) in response to this suggestion.

**8.** *“Very long crosslinks of 50-54Å are observed. It is not clear from the figure how this is possible given that they crosslinked groups are on opposite sides of the structure. It is written that there may be conformational flexibility of L12. L12 is simply a C-terminal tail? What are the possible lysines involved and are they C-terminal to helix 12?”*

We agree with the reviewer that it was difficult to tell from the image provided what parts of the protein and what lysine residues are involved in forming the long green crosslinks. We have changed Figure S5 to illustrate this better. Reviewer 1, Question 1, was also about this issue and we hope that the text from lines 231-237 also addresses the present question.

**9.** “*Toward the top of page 14 it is written ‘M1 proteins are arranged in a helical fashion with their short edges pointing towards the outside of the tube (S9 Fig)’. Can it be clarified what is meant by 'short edges'*?

Thank you. This has been rewritten for clarity on lines 302-312, refering to the dimensions of the M1 monomer as a ‘brick’, which seems to us more apt than previous descriptions in the literature. This discussion can be found in lines 322-327.

“All in all, seen as a single brick in the wall of the V97K oligomer, each M1 monomer displays the edge that contains the V97K mutant on the positively charged outer surface. The dimensions of the M1 bricks (35 by 65 Å; S10 Fig) are consistent with previous negative-stain EM analyses of the matrix layer in intact virions [10,30]. Each turn of this helical wall displays a pitch of 42 Å, in agreement with previous cryo-EM analyses of matrix layers within virions (Table 1) [6].“

**10**. “*I find the following text in the legend to Figure 4 confusing: "Identified intramolecular DSS crosslinks of M1 within oligomers from purified protein (upper panels), within spherical PR8 virions (middle panels) and within filamentous Udorn virions (lower panels)". And also the label ‘Oligomers’ in the Figure 4C. Presumably all of these are oligomers, including those from virions?*”

Thank you, we have broken up the very long sentences in the figure legend and changed the explication of Figure 4C in lines 426-437.

“Identified intramolecular DSS crosslinks of M1 within oligomers from purified WT-M1 and M1-V97K proteins are shown in the upper panels. Middle panes show DSS crosslinks of M1 within spherical PR8 virions. Lower panels show DSS crosslinks within filamentous Udorn virions.“

**11.** “*Typos: Fig. 7F 'WT-M1 olgigomers'; Line 3 of figure 2 legend.”*

We thank the reviewer for pointing out these things. We have corrected them.
